# Mechanisms of stochastic onset and termination of atrial fibrillation studied with a cellular automaton model

**DOI:** 10.1098/rsif.2016.0968

**Published:** 2017-03-29

**Authors:** Yen Ting Lin, Eugene T. Y. Chang, Julie Eatock, Tobias Galla, Richard H. Clayton

**Affiliations:** 1 Theoretical Physics Division, School of Physics and Astronomy, University of Manchester, Manchester, UK; 2 Department of Computer Science and INSIGNEO Institute for in silico Medicine, University of Sheffield, Sheffield, UK; 3 Department of Computer Science, Brunel University London, Uxbridge UB8 3PH, UK

**Keywords:** atrial fibrillation, re-entry, termination, cellular automata, model

## Abstract

Mathematical models of cardiac electrical excitation are increasingly complex, with multiscale models seeking to represent and bridge physiological behaviours across temporal and spatial scales. The increasing complexity of these models makes it computationally expensive to both evaluate long term (more than 60 s) behaviour and determine sensitivity of model outputs to inputs. This is particularly relevant in models of atrial fibrillation (AF), where individual episodes last from seconds to days, and interepisode waiting times can be minutes to months. Potential mechanisms of transition between sinus rhythm and AF have been identified but are not well understood, and it is difficult to simulate AF for long periods of time using state-of-the-art models. In this study, we implemented a Moe-type cellular automaton on a novel, topologically equivalent surface geometry of the left atrium. We used the model to simulate stochastic initiation and spontaneous termination of AF, arising from bursts of spontaneous activation near pulmonary veins. The simplified representation of atrial electrical activity reduced computational cost, and so permitted us to investigate AF mechanisms in a probabilistic setting. We computed large numbers (approx. 10^5^) of sample paths of the model, to infer stochastic initiation and termination rates of AF episodes using different model parameters. By generating statistical distributions of model outputs, we demonstrated how to propagate uncertainties of inputs within our microscopic level model up to a macroscopic level. Lastly, we investigated spontaneous termination in the model and found a complex dependence on its past AF trajectory, the mechanism of which merits future investigation.

## 1. Introduction

Mathematical and computational models have become an increasingly popular tool for investigating biological and physiological systems. The quantitative capabilities of models can provide both unique insights into the mechanism of a problem and predictive power beyond experimental or clinical preparations. Once developed, a model can be used to test and generate future hypotheses in a way that may not be possible in experimental settings. The holy grail of computational biology is to develop comprehensive models that describe both mechanistic properties—for example, detailed molecular dynamics of biochemical interactions in a living organism—and subsequent emergent phenomena.

Development of comprehensive models is constrained by current computational power as well as lack of data. Instead of building comprehensive models, a more adaptable approach is to select a relevant spatial and temporal scale of the phenomenon and devise models that suit a particular research question. Thus, for the same biological or physiological systems, a wide spectrum of models may coexist, which aim to explain and predict the physiological process at different length or time scales. By analysing these models separately, researchers can gain a deeper understanding of the regimes where the different quantitative models are adequate. Bridging these models provides a way to propagate outputs derived from one model into inputs for another model.

In computational cardiac electrophysiology, there exist a range of models, which have been used to examine how subcellular electrical processes influence the diffusion of activation wavefronts across the heart [1]. Computing detailed biophysical models involves solving large systems of coupled ordinary or partial differential equations, which is computationally demanding. This limits both the number of simulations that can be run as well as their duration. It is therefore difficult to explore the sensitivities of a given model to input parameters and initial conditions. This, in turn, means that model outputs cannot easily be translated into inputs for models at other scales, such as those describing progression of patients through care pathways [2].

Atrial fibrillation (AF) is a cardiac arrhythmia that remains poorly understood despite progress in the development of detailed cardiac electrical models, experimental work and clinical studies. AF presents a prevalent heart rhythm disorder that significantly increases stroke risk in humans [3]. Improving identification, management and treatment of AF remains an important challenge [4]. AF consists of episodes of rapid and self-sustaining electrical excitation in the atrium of the heart, which punctuate periods of normal sinus rhythm when activation is driven by the heart's natural pacemaker. As the disease develops, episodes of AF become longer and more frequent until AF becomes permanent. Episode duration can vary between seconds and weeks, and constitutes the basic clinical marker to classify AF progression in patients. The ability to model and predict episode duration for a given patient would therefore be of significant clinical interest. In a previous publication [5], we described a biophysically motivated agent-based stochastic model to simulate progression of AF in a patient from first diagnosis, based on generating a time series of AF episodes with varying durations. The model parameters, which predicted episode start times and durations, were estimated from the literature where possible.

The duration of an AF episode is determined by mechanisms underlying its initiation and termination. AF is thought to be driven by rapid and self-sustaining electrical activity predominantly occurring in the left atrium [4,6]. An important mechanism is re-entry, in which a circulating activation wave continually propagates into recovering tissue. Several mechanisms have been associated with re-entry initiation, including atrial fibrosis [7], pulmonary vein triggers [6], and action potential and conduction velocity restitution [8]. Meanwhile, mechanisms of AF termination remain a poorly researched area, in part, owing to the computational cost of evaluating complex cardiac electrophysiology models over long periods. Initiating and maintaining an AF episode up to its termination in a simulation of a biophysically detailed model requires significant computational resources [9] if the episode lasts for more than a few seconds and/or a detailed atrial geometry is used.

Cellular automata (CA) models of the electrical activity on the surface of the heart are a simplified representation of cardiac electrophysiology, and the very first computer model of AF was a CA model [10]. They provide an intuitive way of describing how cardiac cells activate (depolarize) and deactivate (repolarize) by using simple update rules for the state of a single cell. These are usually based on the present states of the cell itself, and of its nearest neighbours. Since the original five-state Moe model [10], a series of studies have established that CA models of electrophysiology can represent behaviours seen with more detailed biophysical representations of excitable media [11–14]. CA models are simple to program and computationally cheap to run, allowing large numbers of simulations for little cost, and more detailed CA models have recently been devised [15,16].

The motivation of this study was to use a CA model as a computational platform to investigate stochastic initiation and termination of AF episodes. Our contribution can broadly be summarized as follows. First, in contrast to previous CA models [15,16], which used a simplified geometry such as a two-dimensional sheet with periodic boundary conditions, we generalized to a geometry representing the anatomical topology of the left atrium. While this is still stylized, we think this is a step towards reality. We show that the model is capable of inducing and terminating AF episodes stochastically. These phenomena are in line with the predictions of current state-of-art mechanistic models, and we are confident the CA model captures the essential dynamics of the real physiological system. Thus, we propose that CA models are a reasonable compromise between reality and computational efficiency when large numbers of long duration simulations are required. Second, we present a framework to analyse and infer the rate of stochastic initiation and termination of AF episodes. With the ability to run large numbers of simulations over long durations, we were able to accurately quantify these rates. This is necessary in order to be able to predict—in a statistical sense—the future progression of patients at a longer time scale. For example, these rates can be used to connect the CA model to the model we proposed to represent AF progression over years and decades [5]. We propose a framework of statistical analysis of patient trajectories, and apply it to a set of patient trajectories, generated from the CA model. We believe the ideas suggested may also be applicable to data from mechanistic models of other physiological systems, when computational resources are available to generate sufficiently many sample paths from such models.

## 2. Methods

### 2.1. Model geometry

Electrical activation was modelled on an idealized spherical geometry, representing the left atrium of the human heart. We did not include the right atrium, because the main drivers of AF are believed to originate in the left atrium. In order to approximate the dimensions of the human left atrium, the volume of the sphere representing the left atrium was set to 40 ml [17], corresponding to a radius of 21.2 mm. We rescaled this geometry to obtain a unit sphere.

**Figure 1.**
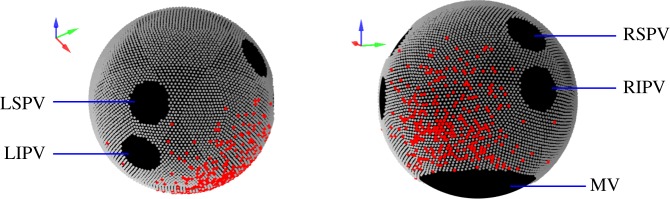
Visualization of the spherical geometry representing the left atrium, with nodes distributed regularly over the surface. Anatomical features (black) were rendered electrically inactive. LS/LIPV, left superior/left inferior pulmonary vein. RS/RIPV, right superior/right inferior pulmonary vein. MV, mitral valve. Fibrotic cells (red) were distributed randomly over a disc centred on the posterior atrial wall.

We implemented a Moe-type CA [10] in which the dynamics take place on discrete nodes on the surface of the sphere. To place the nodes on the spherical surface as uniformly as possible, we used an icosahedral dissection [18] to distribute 10 242 points evenly on the sphere, as visualized in figure 1. We also investigated an alternative way to distribute nodes using an Archimedian spiral [19]; this method can be generalized to non-spherical surfaces.

Polar coordinates (*θ*, *ϕ*) were used to specify the locations of the nodes. We defined the anterior and posterior direction to be (*θ*, *ϕ*) = (*π*/2, 0) and (*π*/2, *π*), respectively.

On the geometry, the anatomical objects—four pulmonary veins (PVs) and the mitral valve (MV)—were set to be electrically inactive. The MV, modelled as a circular area centred on the south pole (*θ*, *ϕ*) = (*π*, 0), was estimated to have circumference of 85 mm [20]. The four PVs were modelled as circular areas with a base radius of 5 mm [21] corresponding to 0.236 scaled units. The PVs were placed at (*θ*, *ϕ*) = (2*π*/5±*π*/10, ±*π*/3). Nodes in these areas were permanently removed, and the remaining nodes comprised the substrate for the CA to take place.

#### 2.1.1. Fibrosis

Fibrosis on the posterior atrial wall is thought to play an important role of inducing AF re-entry [1,7,22], and was modelled by removing nodes in the corresponding area. To model the spatial heterogeneity of fibrosis, we removed nodes according to a probability distribution set to be normally distributed, centred at (*θ*, *ϕ*) = (0.65*π*, 0), with a standard deviation equal to 0.4 sphere radii. The number of nodes removed (denoted FC) quantified the severity of fibrosis. Time-dependent fibrosis was not investigated in this study, as structural modelling of atrial tissue with fibrosis occurs at a time scale much slower than that of re-entrant activity [23].

### 2.2. Dynamics of the cellular automaton model

A multi-state Moe-type CA was used to represent electrical excitation in each node (or ‘cell’). Each node on the sphere could be in one of a number of discrete states, labelled 0, 1, 2, … . In this type of discrete-time model, an action potential is represented by a time delay, during which an excited cell may trigger neighbouring cells within an interaction radius but cannot itself be re-excited. In our model, the cell was deemed ‘at rest’ at state 0 and ‘activated’ if its state was greater than 0. A cell at rest would become excited if the number of ‘recently excited’ neighbours in a local radius exceeded a threshold, upon which it changes from state 0 to state RP, the RP or action potential duration, at the next time step. A neighbour was considered to be ‘recently excited’ if it had been activated in the past four time steps. This number was chosen to achieve realistic spread of excitation (see below). Following excitation, the activated cells reduced their state by one each time step until the state reached 0, i.e. the ‘rest’ state. Each discrete time step in our simulation corresponds to approximately 2.5 ms in real time. In sinus rhythm, RP took values of about 120 time steps in the model (variations are described below), this representing a physiological RP of 300 ms.

To avoid grid discretization effects on the simulations owing to non-uniformities of the icosahedral mesh, the interaction radius between cells on the sphere was set to be greater than the length scale of the typical internode spacing (for complications, see Ventrella [24]). The speed at which an excitation wavefront could propagate (conduction velocity) was determined by two free parameters: the search radius and the threshold of number of active neighbours. We carefully calibrated both the active neighbour thresholds (= eight nodes) and local search radius (2.544 mm), corresponding to a region containing ≈36 nodes to achieve a baseline conduction velocity across the sphere of 0.5 m s^−1^. Thus, the total time taken to travel across the unit sphere (defect-free) from north pole to south pole was ≈133 ms.

#### 2.2.1. Sinus rhythm

The sinus node (SN) is located in the right atrium, so in our model, sinus rhythm was represented by the regular activation of a region of cells proximal to the right pulmonary veins (a circular area centred at (*θ*, *ϕ*) = (5*π*/12, *π*/2) with radius 1.696 mm), which is typically the site of earliest activation in the left atrium following right atrial activation. The sinus period was set at 1 Hz for all simulations, unless otherwise specified.

#### 2.2.2. Pulmonary vein triggers

Bursts of spontaneous activation near the PVs are thought to be triggers of re-entry [6]. To model PV bursts, a 2 mm annulus around each of the four PVs was set to be capable of autoexcitation. In each time step with probability *p*, one node in these annulus regions and its surrounding nodes (set as those points within 2.12 mm to the selected node) spontaneously fired to its maximal state. The location of this spontaneous firing was chosen uniformly on the annuli. The probability, *p*, quantifies how often these bursts occur; the corresponding burst rate in a continuous-time setting can be computed using *p*/(time step) = continuous-time bursting rate BR, which is set as a model parameter. Note that triggers were stochastic and occurred *on average* BR times per second, rather than occur periodically every one per BRs.

#### 2.2.3. Restitution

To model the effect of *restitution* where the RP (i.e. action potential duration) of a cell shows sensitivity to its previous rate of excitation, we implemented the following (non-dimensionalized) formula [25]2.1
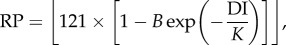
where DI is the diastolic interval (the quiescent interval between the end of one activation and the following beat), and *B*, *K* are parameters controlling the steepness of the curve. *K* had units of discrete time (=2.5 ms), and *B* was dimensionless. The floor function enforced that RP was an integer, which in combination with scale factor 121, allows a maximal RP of 120 time steps. RP was subject to a minimum of 64 time units, i.e. RP = max(equation (2.1),64). This equates to 160 ms (considered the shortest physiologically relevant RP). We investigated the dependence of the transition rate into AF episodes on parameters *B* and *K*.

### 2.3. Implementation

The model was implemented with custom code written in C++, and is publicly available on Github at https://github.com/dblueeye/atrial-fibrillation-cellular-automata. Links to sample movies may also be found. The simulation ran at 16× speedup, i.e. 16 s of simulated time could be evaluated in 1 s. The skeleton code of the simulation is detailed below to clarify implementation steps:

(1) Initiation: set up location of the nodes on the sphere. Remove nodes on areas of the PV and MV. For each sample run, model fibrosis by removing a fixed number of nodes according to a spatial probability distribution. Briefly, assign a probability to each node, generated from a normal distribution centred at (*θ*, *ϕ*) = (0.65*π*, 0) with a standard deviation 8.48. Then, arrange the probabilities into a list and compute the cumulative probability distribution *F*(*i*) with respect to the list. The inverse transform sampling was applied to the discrete distribution to select the node to be taken out. Repeat the procedure until FC numbers of nodes were taken out. Generate and store the nodes representing SN breakthrough. Generate and store a list of possible PV bursting locations and the nodes which would burst in a group. Generate a neighbourhood map between the nodes.
(2) SN breakthrough: check if in this time step SN breakthrough occurs. If so, activate the nodes of SN to their maximal state as follows: using the cycle length (CL; time between SN pacing) and RP from the previous cycle, compute DI = CL − RP. Use equation (2.1) to compute and update the RP of this node, and activate its state to RP. If in this time step SN breakthrough does not occur, the state of SN breakthrough nodes is reduced by 1.
(3) PV bursts: with probability *p*, there will be a PV burst. If this happens, choose one of the locations where PV bursts can take place. As described above, a group of nodes in that region is activated to their maximal state, and the new RP is computed and updated using equation (2.1).
(4) Rest of the nodes: for the remaining nodes, check if any neighbours in the interaction range have been activated in the past four time steps (10 ms). If so, this node is activated to its maximal state, RP is again updated according to equation (2.1). Otherwise, the state of the node is reduced by 1.
(5) Repeat from 1 until end of simulation.

## 3. Results

### 3.1. Stochastic initiation of atrial fibrillation

**Figure 2.**
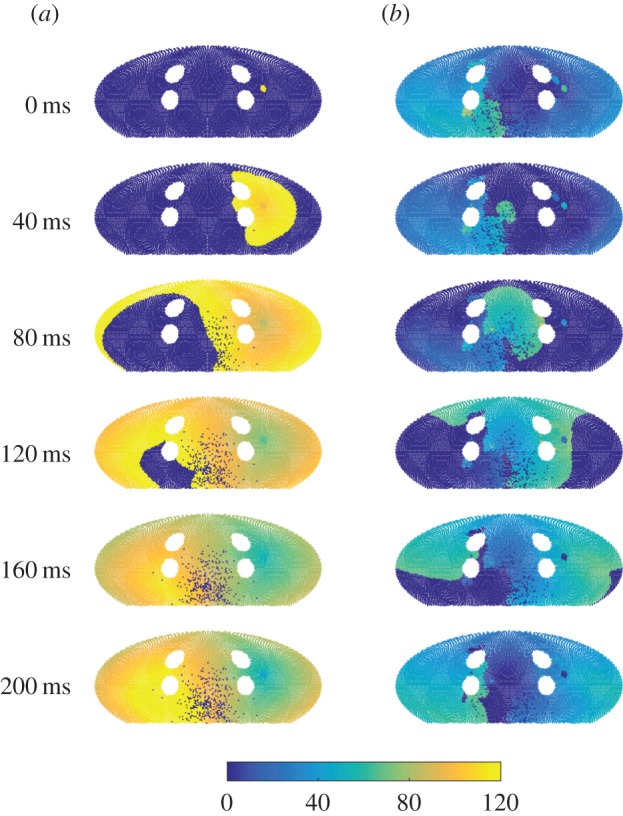
Snapshots of the simulation using a Mollweide projection. The centre of the projection is at the posterior atrial wall. Fibrotic nodes (red), which cannot be initiated, are set to a constant state. (*a*) In sinus rhythm, SN breakthrough starts proximal to the right pulmonary veins (PVs), and cells are immediately excited from 0 to (maximal refractory period, RP) state 120, decreasing its state by 1 each time step until it reaches 0. Cells nearby are excited to 120 if the number of neighbouring cells which are excited exceeds 8, and this starts a wavefront of activation over the sphere, with slower activation through fibrotic areas and around PVs. (*b*) In re-entry, existing wavefronts self-perpetuate across the domain, and SN breakthrough does not initiate wavefronts of excitation. RP restitution has meant that cells excite to a lower state compared with sinus rhythm, and this also leads to a shorter wavetail.

Simulation results were visualized using an equal-area Mollweide projection [26], shown in figure 2. During sinus rhythm without PV bursts (figure 2*a*), activation of the left atrium began by SN breakthrough near the right PVs; wavefronts passed around the larger PVs smoothly with a conduction speed of 0.5 m s^−1^. When wavefronts passed through areas of fibrosis, conduction slowing and conduction block were observed occasionally when the number of activated nearest neighbours remained subthreshold. When PV bursts were introduced, triggers initiated activation near single PVs at a constant rate; in some simulations, this led to transient re-entrant wavefronts forming, and in certain cases, these became permanent re-entrant wavefronts (figure 2*b*). Movies were uploaded to YouTube, and can be found on the Github project page, see the supporting information.

While the complete course of the stochastic process (for each node) could be stored, the resulting data file would be impractically large. Instead, we evolved the CA without exporting the dynamic states at all time steps. As our aim was to investigate statistical properties of the system initiating and terminating AF (defined as self-sustained activity differing from sinus rhythm), two AF classifiers were developed. We stored only the seeds of the pseudo-random number generator of those sample paths, which were classified as ‘in AF’ (details described below). If needed, the collected seeds could recreate the sample paths for subsequent analyses. This procedure permitted generation and storage of up to 10^4^–10^6^ simulation runs, necessary to accurately compute the statistics of AF episodes, including the sampling of rare events such as termination.

### 3.2. Probability of initiating spontaneous re-entry and atrial fibrillation

**Figure 3.**
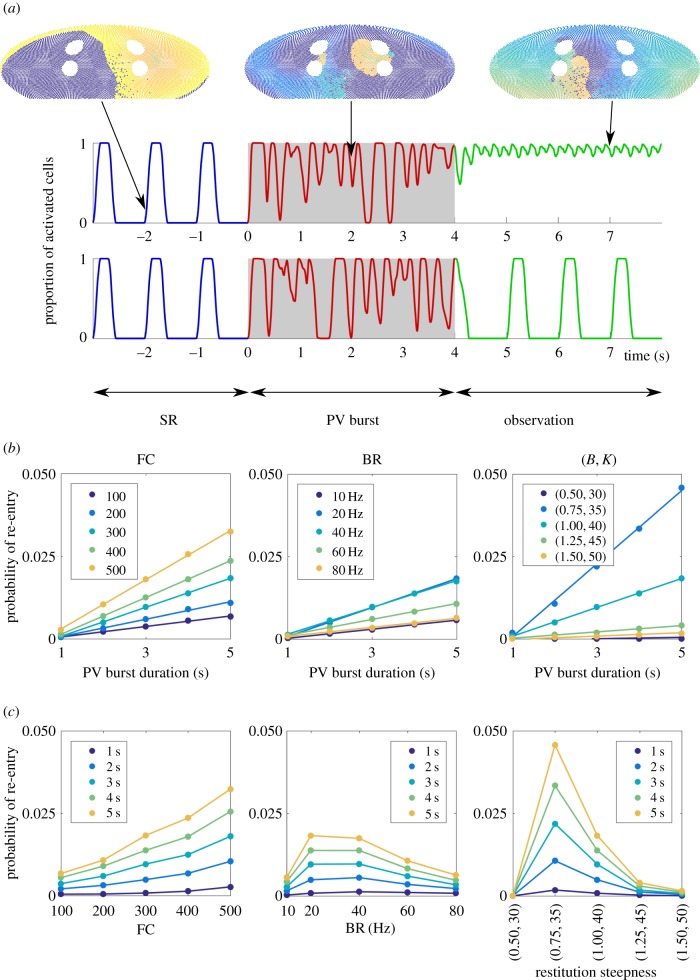
(*a*) Protocol for investigating AF initiation. Starting in sinus rhythm (SR), PV bursts of up to 5 s were initiated, after which a 10 s observation window with sinus pacing was simulated to probe existence of AF. (Top/bottom sample path: with/without AF. In the top sample path, the sinus breakthrough region cannot be excited by sinus pacing, because re-entrant waves keep re-exciting the region from state 0.) (*b*) Probability of re-entry (the proportion of simulations finishing in re-entry over 10^5^ sample paths) as a function of the PV burst duration, for the model parameters fibrosis density (FC), PV bursting rate (BR), and restitution steepness (*B*,*K*). The slope of the curves quantifies the continuous-time rate to induce AF re-entry. Discrete markers: simulation results; continuous lines: best linear fits. (*c*) Data replotted as a function of model parameters, with each line representing PV burst duration, which highlights the non-monotonic dependence on model parameters for BR (middle) and restitution steepness (*B*, *K*). We have joined the scatter plots in this panel for optimal visualization of the non-monotonicity in this panel.

An exploration of the model parameter space was undertaken to determine the primary mechanisms of re-entry initiation. Each simulation was started in sinus rhythm (by setting *p* = 0), then PV bursts of varying time durations were initiated by setting *p*≡BR/400, to simulate PV triggers on the domain. Following a period of time with PV bursts, *p* was reset to 0, and the simulation evolved for a further 10 s observation window (figure 3*a*, snapshots). The proportion of activated cells (in all nodes excluding fibrotic ones) at each time point was tracked, as a simple classifier of re-entry; simulations in which the proportion of activated cells remained non-zero over the entire observation window were deemed in re-entry. An example can be found in the top panel of figure 3*a*; the first time series remained in re-entry, whereas the second time series returned to sinus rhythm. The proportion of sample paths leading to re-entrant wavefronts determined the probability of a given parameter set causing re-entry.

We varied the following parameters: number of fibrotic cells (FC), PV bursting rate (BR) and restitution steepness (*B*, *K*). Baseline simulation parameters were: FC = 300 (points), BR = 20 Hz, *B* = 1.0 and *K* = 40 (discrete-time unit, = 100 ms), and we point-mutated the parameters (FC,BR,*B*, *K*)—see table 1 for the full range. For each parameter set, PV burst duration was varied from 1 to 5 s, and 10^5^ sample paths were generated to compute the probability of inducing re-entry. Results are summarized in figure 3*b*,*c*.

**Table 1.** Numerical results of best fits to the model results using the parameter sets (first column), for simulations up to 5 s using a two state linear transition model (second column), and up to 300 s using a two state nonlinear model (third and fourth columns). *r*_1_ is the rate of AF initiation in both models, and *r*_2_ is the rate at which initiation is inhibited for the nonlinear model. In specific parameter sets, *r*_1_ < *r*_2_ which suggests a very low rate of AF initiation.

parameter (FC,BR,*B*,*K*)	best fit *r*_1_ in figure 3*b*,*c*	best fit *r*_1_ in figure 5*a*,*b*	best fit *r*_2_ in figure 5*a*,*b*
(300, 0.05, 1, 40)	4.395 × 10^−3^	4.095 × 10^−3^	6.796 × 10^−5^
(100, 0.05, 1, 40)	1.603 × 10^−3^	1.567 × 10^−3^	9.838 × 10^−5^
(200, 0.05, 1, 40)	2.646 × 10^−3^	2.569 × 10^−3^	9.377 × 10^−5^
(400, 0.05, 1, 40)	5.558 × 10^−3^	5.106 × 10^−3^	7.403 × 10^−4^
(500, 0.05, 1, 40)	7.451 × 10^−3^	4.553 × 10^−3^	3.562 × 10^−3^
(300, 0.025, 1,40)	1.395 × 10^−3^	1.255 × 10^−3^	2.720 × 10^−9^
(300, 0.1, 1, 40)	4.069 × 10^−3^	4.095 × 10^−3^	6.796 × 10^−5^
(300, 0.15, 1, 40)	2.403 × 10^−3^	2.516 × 10^−3^	1.027 × 10^−8^
(300, 0.2, 1, 40)	1.371 × 10^−3^	1.580 × 10^−3^	5.350 × 10^−4^
(300, 0.05, 0.5, 30)	2.451 × 10^−4^	3.883 × 10^−6^	1.908 × 10^−3^
(300, 0.05, 0.75, 35)	1.108 × 10^−2^	1.125 × 10^−2^	1.123 × 10^−5^
(300, 0.05, 1.25, 45)	9.469 × 10^−4^	8.774 × 10^−4^	1.463 × 10^−5^
(300, 0.05, 1.50, 50)	4.707 × 10^−4^	3.417 × 10^−4^	4.371 × 10^−4^

We found the probability of AF re-entry depended linearly on the duration of the PV bursting when this duration was less than or equal to 5 s. This suggests AF initiation may be modelled by a simple coarse-grained model in continuous time, in which initiation occurs with constant rate, written as follows:3.1
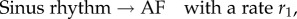
where the transition rate *r*_1_ is the slope of the linear response shown in the left panel of figure 3*b*,*c*. We performed a linear fit to the numerical data, and found the rate was monotonically dependent on fibrosis: *r*_1_ is larger for higher FC. Estimated values for *r*_1_ are reported in table 1.

We found a non-monotonic relation between re-entry probability and PV burst rate BR, seen in the middle panels of figure 3*b*,*c*. A PV burst was most likely to induce AF when BR was between 20 and 40 Hz. This could be due to ‘crowding’ effects in the case of BR, reducing excitable regions or increasing the likelihood of wavefront collision and termination of re-entry. Similarly, the rate into re-entry had a non-monotonic response to the restitution parameters, increasing AF initiation rate then decreasing as the parameters were increased, steepening the restitution slope, as shown in the right panels of figure 3*b*,*c*. For increased restitution steepness (larger (*B*, *K*)), a potential mechanism could be that increased spatial heterogeneity of RPs is mediated by shorter refractory tails. These observations suggest that the CA model is able to capture complex interplay between the mechanisms inducing AF.

### 3.3. Estimating time of atrial fibrillation initiation using a dynamic classifier

In the previous section, we investigated the hypothetical case where we controlled PV burst duration independently and subsequently observed for AF. In reality, PV bursts occur at random and cannot be simply turned off physiologically—AF may have initiated before the end of the burst period. Thus, the previous classifier is insufficient for estimating the true time of AF initiation. An alternative classifier to observe, record and track re-entry was thus proposed to estimate AF initiation time.

To model this, we again used the proportion of activated cells to be our ‘signal’, and defined an alternative AF classifier: tracking the proportion of activated cells out of total (non-fibrotic) cells, above a non-zero threshold for a period of time. We considered this analogous to clinical monitoring methods such as the electrocardiogram, which detect absence of regular peaks (e.g. P waves) for defined periods. Similar methods have been adopted by Manani [16]. In the following analysis, we set the non-zero threshold to be 0.5 and the time period to be 2 s. Using this definition, the classifier operates without perturbing the CA, and the onset time of AF re-entry is a random variable: in different sample paths, the first time the classifier is triggered is random.

**Figure 4.**
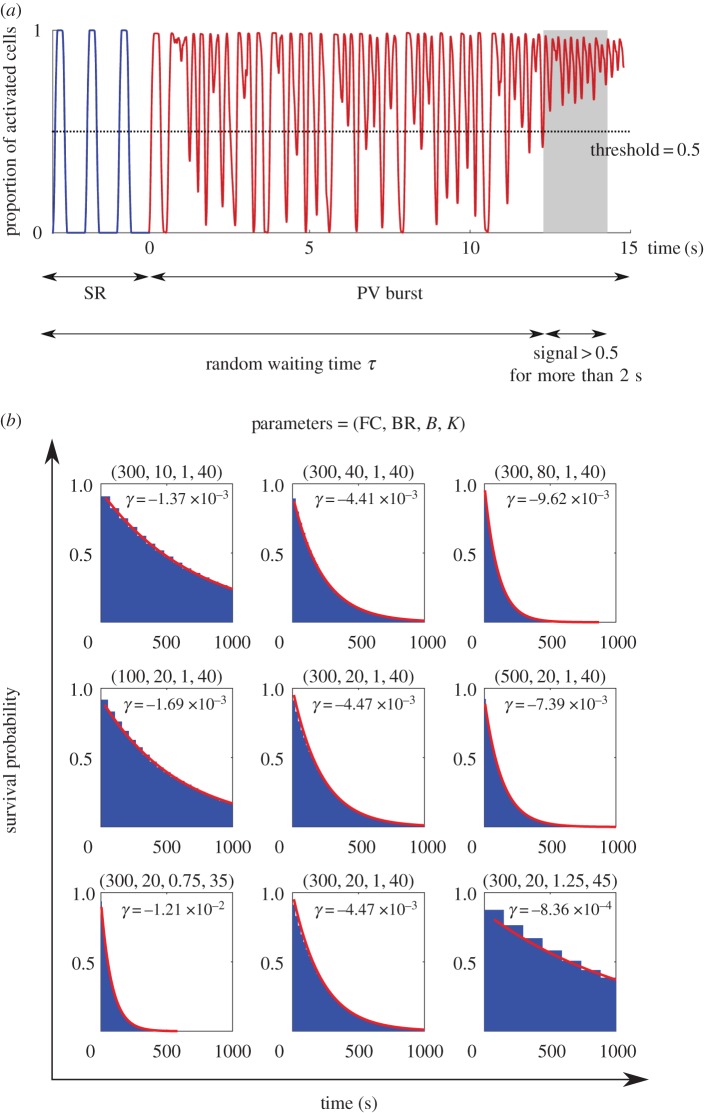
(*a*) The second classifier identifies the system as ‘in AF’ if the proportion of active cells is greater than 0.5 for more than 2 s. (*b*) The onset time of AF is a random time *τ*, and the cumulative distribution of *τ* is plotted for selected parameter sets. The data were fitted by an exponential function 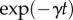. Note that when we varied BR, the cumulative distribution was monotonically decreasing for any given time. This indicates that the second classifier identifies the transition rate to enter AF is a monotonic increasing function of BR, in contrast to figure 3 where AF is mostly induced at the intermediate regime of BR.

We refer to the first time the classifier indicates AF as *τ*. This differs from simulation to simulation, and is random. We simulated 10^5^ samples for selected sets of parameters to compute the cumulative distribution function *s*(*t*) = Prob[*τ* > *t*], the probability that the classifier is not activated before time *t*. This monotonically decreasing function quantified the statistics of the random transitions into the first re-entrant episode: the quicker the cumulative distribution function decays, the faster the system transitions to AF on average. The results are presented in figure 4. The numerical results suggest that the cumulative distribution function is exponential, a signature that the waiting time distribution is also exponential, confirming the simple coarse-grained model with constant rate in equation (3.1).

This analysis suggested a monotonic relation to all parameters, which differs from the first classifier, where burst rate BR showed non-monotonic dependence. We observed that the increased number of PV bursts at high bursting rates raised the proportion of activated cells, triggering the second classifier, but without leading to AF under the definition of the first classifier. To test this observation, we performed the following simulation: after the second classifier identified a re-entrant episode, we turned off the PV bursts and evolved the system for another 10 s. We excluded the sample paths which did not exhibit re-entry at the simulation endpoint, following the first classifier. For all parameter sets except the high BR = 80 Hz case, more than 94% of re-entrant episodes identified by the second classifier led to self-perpetuating re-entry. In the BR = 80 Hz case, only 16% led to re-entry.

This observation showed the second classifier, albeit realistic in practice, overestimated the transition rate into AF.

### 3.4. Longer durations of pulmonary vein bursts suggest existence of spontaneous atrial fibrillation termination dynamics

Using the analysis described above, we obtained a quantitative estimate of time scales for transition to AF. Taking the baseline parameter set (FC, BR, *B*, *K*) = (300, 20, 1, 40), both classifiers estimated an ≈5 × 10^−3^ s^−1^ transition rate into AF; in other words, sinus rhythm is maintained under the influence of PV bursts for ≈200 s. In addition, we would expect all sample paths to transit into AF if we waited long enough.

**Figure 5.**
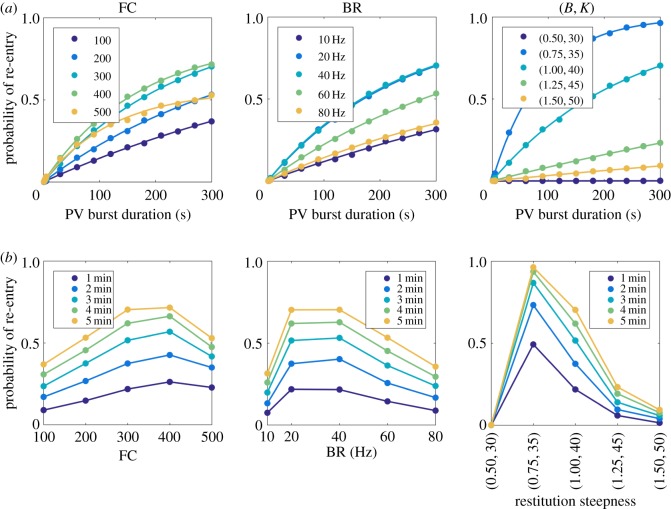
A parallel analysis of figure 3 to analyse AF initiation rate for longer duration of PV bursts up to 300 s. (*a*) Re-entry probability as a function of PV burst duration, for different model parameters. Discrete markers: simulation results; continuous curves: best fits using equation (3.3). (*b*) Re-entry probability as a function of model parameters, for different PV burst durations. Re-entry probability is nonlinear for all parameters, and exhibits non-monotonic dependence on BR and restitution steepness. We have joined the scatter plots in this panel for optimal visualization of the non-monotonicity in this panel.

To test this assertion, we extended the analysis in figure 3*b*,*c* with a longer PV burst duration. For each parameter set, we simulated 5000 sample paths to compute the probability that the sample had transitioned into AF. The results are shown in figure 5. For some parameter sets, after a long period of PV bursting, the probability did not converge to 1.0 (e.g. for FC = 500). In other words, the coarse-grained model equation (3.1) did not sufficiently capture AF dynamics when the PV burst duration was increased.

We thus generalized the coarse-grained model into a two-state model with a stochastic initiation and termination of AF under conditions of PV bursting, where SR represents sinus rhythm:3.2a
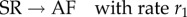
and3.2b
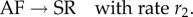


Because we start in sinus rhythm, the initial probability of AF at *t* = 0 is always 0 (and 1 for sinus rhythm). The temporal behaviour of the probability to be in AF can be calculated using standard methods [27], and we find3.3
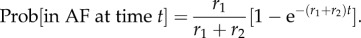
A two-parameter fit was performed for each simulated parameter set, and the best fit is displayed in figure 5. Corresponding values of *r*_1_ and *r*_2_ are reported in table 1. The value *r*_2_ quantifies the time scale at which stochastic AF initiation is inhibited by constant PV bursts. Comparing the relative values *r*_1_ and *r*_2_, with high FC or low BR, inhibition of AF initiation dominated the process (*r*_1_ < *r*_2_) and the response of the termination rate to the parameters was also non-trivial.

### 3.5. Estimates of spontaneous atrial fibrillation initiation and termination times

To propagate results to our previous model of long time scale AF progression [5], we attempted to project a two-state stochastic model to predict progression of AF at longer time scales. Physiologically, PV bursts occur in acute time periods (≲1 s [6]) rather than occur chronically. To model this phenomenon, we proposed the following two-stage and two-state model:3.4a
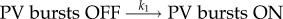
and3.4b
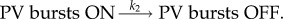
When PV bursts are in ON state,3.5a
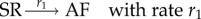
and3.5b
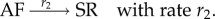
Otherwise, the state of the system remains in SR/AF, respectively. Here, 1/*k*_1_ and 1/*k*_2_ quantify the average duration of the resting state (no PV bursts) and active state (with PV bursts), respectively. Short trains of bursting mean that *k*_2_ ≫ *k*_1_. Selected parameter regimes were tested (data not shown) and preliminary results showed the coarse-grained model equations (3.4) and (3.5) faithfully projects the progression of the CA model for a range of parameter regimes. However, at much longer time scales 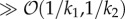, there were notable discrepancies. We investigated these differences in the following section, which suggests existence of higher-order states of AF dynamics.

### 3.6. Fourier analysis revealed higher order dynamics of atrial fibrillation

**Figure 6.**
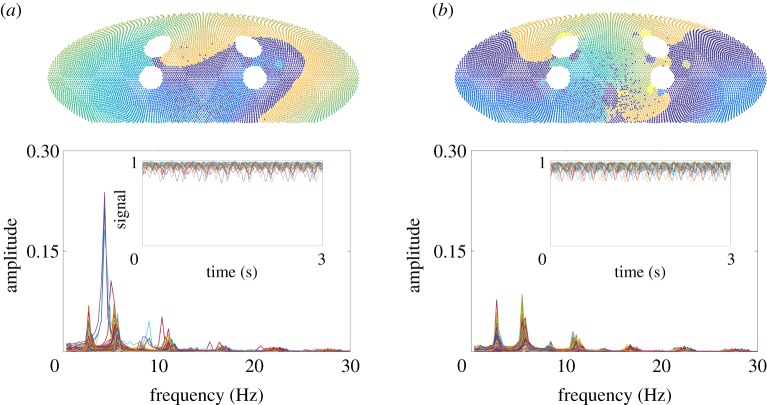
Fourier analysis of the classifier signal in AF re-entry. Both panels used baseline parameters FC = 300, BR = 20 Hz, *B* = 1 and *K* = 40. In (*a*), the Fourier spectra of 200 sample paths ending with re-entry after 5 s PV bursts were overlaid. In (*b*), 200 sample paths with re-entry after 150 s PV bursts were plotted. We plotted 20 sample paths on the temporal domain in the insets. Above the main plots, we present typical snapshots of the visualization (movies provided in the supporting information), showing a more ‘homogeneous’ travelling wave in (*a*), and a more ‘fragmented’ wavefront in (*b*).

Our numerical simulations yielded many (≳10^4^) sample paths which ended in AF re-entry. Fourier analysis was applied to sample paths from the baseline parameter set where re-entry was initiated (corresponding to the ‘observation’ phase of the time series shown in figure 3*a*, top panel). Results for 200 sample paths are shown in figure 6.

For 5 s PV burst duration (figure 6*a*), we visualized sample paths along with the Fourier analysis, and observed that the dominant mode ≈5 Hz corresponds to the period of a single re-entrant wavefront. Subdominant half modes ≈2.5 Hz corresponded to the period of points that experienced 2 : 1 conduction block, e.g. points near one of the PVs which have previously been fast paced. There also existed higher harmonics, to which we did not seek to fit a physiological interpretation.

There was a notable variability in the Fourier spectrum for each sample path. This reflected the stochasticity of the system—including the quenched heterogeneity of RP, fibrosis and dynamical randomness from PV bursts—which propagated to the dynamics of re-entry modes. As the speed of the travelling wave is fixed at conduction speed 0.5 m s^−1^, the dominant frequency is inversely proportional to the path length the wavefront travelled in one cycle. Both the duration of the re-entry and the length of cycle path exhibited ≈20% variability. We also examined the case when PV bursts lasting 150 s were applied (figure 6*b*), observing that the variability of the spectrum appeared smaller compared with the 5 s case. This suggests longer duration of PV bursts tend to drive the system into a stable dynamical mode that is hard to perturb. By comparing visualizations alongside the Fourier spectrum, we also identified that multiple 2 : 1 conduction blocks formed more frequently, and higher-order rotors were identified. Two representative snapshots are presented in figure 6*a*,*b*, top panel.

This analysis shows that even when the model state was classified as ‘in AF’, there can be multiple modes. The follow on question is whether the complexity of an AF episode affects its stability and its likelihood to terminate, either spontaneously or following intervention. For a single re-entrant wavefront, a short PV burst at the right time and location terminated AF (movie on YouTube, see the supporting information). This led to an investigation into spontaneous termination of AF in §3.7, comparing termination rates for different AF modes, to infer likely mechanisms of termination.

### 3.7. Investigation into stochastic atrial fibrillation termination suggests stable and unstable re-entry modes

**Figure 7.**
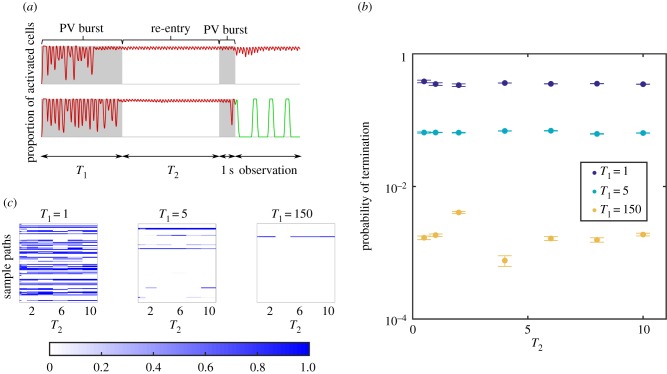
(*a*) Schematic diagram of the protocol to probe spontaneous termination. We turned on PV bursts for a duration *T*_1_ (unit: s), to obtain sample paths which initiated AF. For these, a second PV burst of duration 1 was applied, following a waiting window of duration *T*_2_ (s). Termination probability was measured in the 10 s window after the second PV burst. (*b*) The termination probability across all sample paths depends on *T*_1_; in contrast, the value of *T*_2_ does not change the termination probability significantly. (*c*) We performed 50 trials for *T*_2_ = [0.5, 1, 2, 4, 6, 8, 10], for each sample path in AF after the first PV burst. Probability of AF termination for each sample path (single line), as a function of the sample path and *T*_2_, is shown in the heat map. Strong correlation in the horizontal direction suggests two subpopulations: stable AF which cannot be terminated, and unstable AF which can be terminated, regardless of the *T*_2_ value. The results suggest the subpopulation of stable AF increases as *T*_1_ increases.

Observation of simulations which generated figure 5 indicated stochastic termination of AF could be a direct result of PV bursts. To test this hypothesis, we randomly collected 500 sample paths ending in AF in previous experiments and performed 50 extended simulations on each. Recall that AF was induced by a set of PV bursts over some duration, say *T*_1_, in previous experiments. After AF was initiated, we waited a time window *T*_2_ without PV bursting, and applied another set of PV bursts (1 s duration), and observed if re-entry was terminated after the second set of PV bursts had been applied. A schematic diagram of this is shown in figure 7*a*.

Figure 7*b* shows the termination probability significantly depends on *T*_1_. For *T*_1_ = 1 s, it was very likely to terminate AF, with probability approximately ≳0.3, and it was independent of *T*_2_. For *T*_1_ = 5 s, termination probability was of order ≲0.1, and for *T*_1_ = 150 s the probability went down to order 10^−3^.

This analysis suggests two modes of AF: some re-entrant circuits can be terminated easily by PV bursts, and others cannot. In figure 7*c*, we show probability to termination, ordered by each sample path in the *y*-direction and each waiting window duration *T*_2_ in the *x*-direction. A clear alignment in the *x*-direction of either blue or white stripes showed that if a sample path can be terminated, the probability of termination does not critically depend on *T*_2_; if the sample path cannot be terminated, most likely, it cannot be terminated for any *T*_2_. The longer *T*_1_ (the duration of the first set of PV bursts to induce re-entry), the smaller the proportion of unstable AF (episodes which can be terminated). Thus, the overall probability to terminate AF is orders of magnitude smaller than AF induced by shorter *T*_1_.

We therefore hypothesized that activation and termination can be modelled using the multiple-state model:3.6a
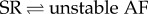
and3.6b
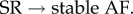
Results suggest that the transition rates are not constant and critically depend on the duration of PV bursts. To quantify transition rates, a classifier identifying the signal state must be developed; we aim to develop this in the future. Our presented framework can be applied to measure transition rates once a reliable classifier is implemented. We remark that the multiple-state system has a ‘memory’ for marginal observables (in AF or not) in line with our previously proposed hidden state binary model [5], which can be used to project the progression of AF over long time scales.

## 4. Discussion

In this study, we investigated stochastic onset and termination of AF episodes by using a CA model on a two-dimensional sphere, with an equivalent topology of the human left atrium. We demonstrated the capability of the model to generate large sets of sample paths to infer the statistical properties of initiation and termination of re-entry and AF (up to 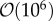 sample paths and for duration approximately 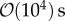). Three potential arrhythmogenic mechanisms were investigated, fibrosis density (FC), pulmonary vein bursting rate (BR) and RP restitution steepness (*B*,*K*). By probing this parameter space, we investigated the probability of AF onset and termination resulting from PV bursts.

We found a linear dependence between burst duration and probability of re-entry initiation for all parameters for short PV burst durations. Increased FC led to a linear increase in probability of initiating re-entry, but there was a non-monotonic relationship between probability of initiating re-entry and both BR and restitution steepness. One possible explanation is that while increased BR and steeper restitution both act to promote re-entry, these mechanisms may also act to promote the termination of re-entry by wavefront collision. When PV burst duration was increased, probability of re-entry at simulation endpoint did not increase linearly, such that at high FC and high BR, likelihood of re-entry remained constant. The behaviour of the model depends on parameters in a non-trivial way, which suggests the existence of complex mechanisms that inhibit or suppress AF initiation, and may even terminate re-entrant circuits before they have fully formed. By fitting a two state nonlinear model to our simulation outputs, we estimated initiation and inhibition rates *r*_1_ and *r*_2_ for given parameter sets.

Finally, we analysed a subset of the sample paths in AF, and found existence of stable and unstable AF modes. A second set of PV bursts could spontaneously terminate a proportion of induced AF episodes, with termination probability reducing, subject to duration of the first PV bursts.

This study offers an alternative novel methodology and framework for investigating mechanisms of spontaneous AF, which differ from conventional modelling and experimental studies in its capability for rapid statistical sampling of longer duration episodes. Our findings and conclusions are set out and discussed in the subsequent paragraphs.

### 4.1. Cellular automaton models of cardiac electrophysiology

Although CA models have been superseded in popularity by more biophysically detailed models [28], CA models show similar tissue scale behaviours [11–14], and thus are still valuable both in stand alone theoretical studies and combined with clinical investigations [15,16,29,30]. Our approach complements studies by Manani *et al*. [16], who used a CA formulation to investigate the effect of time-dependent fibrosis on arrhythmia susceptibility. Our model represents variability and uncertainty through its stochastic formulation and the large number of sample paths, and thus permits a systematic investigation within the model framework, while accepting model limitations. A major limitation of the CA model compared with continuum models is its inability to directly model the mechanism of conduction slowing and CV restitution, although most other potential AF mechanisms [31] may be handled with the CA formulation.

### 4.2. Model parameter space

In this study, we fixed the size and shape of the left atrium, and the size and location of anatomical objects. We did not include the left atrial appendage, and assumed that location of SN breakthrough into the left atrium was fixed. Heterogeneity was investigated by randomly varying initial RPs, rather than by region specific heterogeneity in parts of the left atrium. We recognize that these are all parameters which may vary between individuals, and may significantly impact probability of AF initiation and termination. We chose to focus on biophysical mechanisms rather than on anatomical variability, and recognize that not only is the potential parameter space vast, but also that additional investigations into the effects of these parameters are important.

### 4.3. Atrial fibrillation onset

Recent studies have investigated mechanisms related to electrical and structural remodelling, highlighting the importance of inter-patient variability. McDowell *et al.* [22,32] found that combinations of fibrosis subtypes were proarrhythmic and that patient-specific distribution of fibrosis had a major impact on AF initiation, and anchored wavefronts to specific atrial regions, with other electrophysiological changes not significantly altering this behaviour. Krummen *et al.* [33] reported that steepening AP restitution slope in patients initiated re-entry, with the associated computational study identifying specific ionic pathways responsible for restitution steepening. Regional electrical heterogeneity of the atria was investigated by Colman *et al.* [34], who found region-dependent action potential duration heterogeneity in the atrium increased susceptibility to AF onset and maintenance of re-entrant circuits.

Our study has investigated these three mechanisms plus PV firing rate, albeit with a discrete rather than with continuous model, and different assumptions and formulations (we did not model fibrosis subtypes or include region-specific RPs for our cells). Our study results differ from the conclusions of these continuum studies, especially regarding the steepening of restitution slope, where we found a non-monotonic relationship between AF onset and restitution steepness not predicted by Krummen *et al*. There is no general consensus on whether a steep restitution slope is pro- or anti-arrhythmic [35], and our results showed there is a ‘window’ of steepness which maximizes probability of AF onset. This was also true for the other parameters, where excessive fibrosis and PV burst rate inhibited increased onset of AF. We comment that a PV burst rate up to 80 s^−1^, while representing the number of triggers across all four PVs rather than a single focal source, may appear unphysiological, but it is also possible that many focal PV bursts go undetected.

### 4.4. Atrial fibrillation termination

Clinical studies predominantly investigate how targeted ablations terminate AF, and these have been explored theoretically in a number of studies [36]. However, few studies explore spontaneous termination owing to the difficulty of capturing such rare events. A few clinical studies have been documented: Ndrepepa [37] referred to generators of fibrillatory activity in the left atrium, and reported that AF termination was polymorphic in its mechanism. Alcaraz [38,39] analysed the atrial activity of patients during AF and immediately prior to termination, and found the existence of more organized atrial activity (measured by sample entropy) 1 min prior to termination, and that the late activity had a significantly lower dominant frequency mean value. Some studies of dominant frequency and harmonics have suggested Fourier analyses as useful predictors of termination [40].

Our study was inconclusive regarding termination. We found that PV bursts are a potential mechanism for terminating as well as initiating AF, and also act to inhibit initiation rate for longer durations of PV bursts. Fourier analysis of the sample paths revealed both stable and unstable modes of AF, but no clear trend was observed. We found, however, that the longer the period of PV bursting, the smaller the probability that induced AF will be terminated by future PV bursts. This suggests dynamical memory effects exist within the model caused by extended PV burst pacing, which influences the stability and robustness of the induced re-entry wavefronts. This agrees with the ‘AF begets AF concept’ [41], and recent studies of Uldry *et al.* [42,43], who reported an increase in AF complexity with duration, and that spontaneous termination mechanisms differed depending on dynamics of AF and its underlying complexity.

In other recent studies, Krogh-Madsen *et al.* [44] also found that remodelling maintained AF by shortening atrial wavelength (electrical by shortening action potential duration, structural by slowing conduction), which correlated with increased AF episode duration, with dynamics of re-entry differing between types of remodelling. Liberos *et al*. [9] suggested that cell–cell ionic differences as a mechanism of AF termination, by decelerating re-entrant activity and increase in rotor tip meandering. Our model did not include electrical remodelling similar to these studies, but our model is well placed to analyse atrial wavelength and track the rotor tips in future studies, to see if similar mechanisms exist within our formulation. The general consensus is that AF complexity increases over time together with AF episode durations, with size of atria and atrial obstacles thought to play a critical role in termination. Petrutiu *et al*. [45] found that non-terminating episodes exhibited larger dominant frequencies compared with spontaneously terminating episodes, and more abrupt changes in dominant frequency were observed prior to spontaneous termination. An open mechanistic question remains over whether spontaneous termination is preceded by a progressive fusion of wavelets or a simultaneous block of all wavelets in the tissue. We believe our work is well placed to evaluate these questions through the capabilities to run longer duration simulations.

Our study may additionally complement existing ablation-based termination studies by identifying similar mechanisms or proposing novel therapeutic studies. Rappel *et al*. [46] demonstrated that ablation caused termination in a heterogeneous domain by creating an excitable gap, dislodging a stable anchored wavefront or by closing critical isthmus channels. Uldry *et al*. [47] reported 10–20% success rate when using atrial septal pacing at alternating frequencies to pass the atria.

### 4.5. Clinical relevance

We have presented a methodology to translate emergent simulated behaviour in a greatly simplified computational model of atrial electrophysiology into transition rates into and out of AF. Determining rate transitions between sinus rhythm and AF is an important step forward because these rates can be used in models of AF progression [5], can be evaluated against clinical data, and in the future could be used to predict AF onset and progression.

### 4.6. Future work

Our work in this article focuses on the framework of the stochastic analysis. We acknowledge that CA models are a simplified representation of reality. However, this approach permits large numbers of simulations to obtain probability distributions and probe particular mechanisms. We propose several areas in which the model could be investigated further.

#### 4.6.1. Geometry

We adopted a simplified quasi-spherical geometry to model the left atrium. Because the dynamical rules of the CA only involve the neighbourhood relations between nodes, it is straightforward to construct a CA model on any two-dimensional surface embedded in three-dimensional space. The difficulty of evenly distributing the nodes may be overcome by using the Archimedean spiral [48]. It may also be possible to extend this to three dimensions.

#### 4.6.2. Directional fibrosis

In this work, we modelled fibrosis by setting nodes to be electrically active, whereas fibrosis may act to promote faster propagation in certain directions within cardiac tissue [16]. This could be achieved in our CA model by assigning weights to neighbouring nodes.

#### 4.6.3. Representing interventions

As the computational cost of a CA model is lower than that of biophysically detailed models, it is an ideal platform to develop and evaluate effects of intervention strategies such as ablation or external pacing. However, as a coarse-grained approach, the CA model is unlikely to capture detailed biochemical or biophysical effects within these, or within other interventions such as pharmacological modification of cell and tissue electrophysiology.

#### 4.6.4. Restitution and remodelling

Restitution changes may not be instantaneous. One way to model restitution with memory would be to replace equation (2.1) by4.1
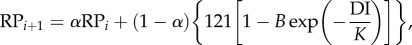
where *α* measures the strength of the ‘memory’. When *α* = 1, there exists no restitution, and when *α* = 0, it converges to our proposed model (2.1).

Only initial state structural remodelling was investigated in this study. Additional structural and electrical remodelling may be implemented in the CA framework, both as an initial condition and as a transient process (e.g. with ageing). For example, removing cells (and adding them back) from the domain of excitable cells could model acute scar formation or recovery from ischemia.

#### 4.6.5. Pattern recognition of the re-entrant wavefronts

Our analysis revealed stable and unstable modes of AF. Visualization of selected sample paths suggested some characteristic differences between these modes: just prior to termination, unstable AF terminated via conduction block through fibrosis regions or pulmonary veins. This often included spontaneous PV bursts at the channel isthmus in a short excitable window. In comparison, stable (did not spontaneously terminate) wavefronts appeared to have more complex pathways of propagation.

While the Fourier spectrum suggested potential differences, the analysis was inconclusive as there was a large variability over sample paths in a given parameter set. As our classifiers contain only the temporal information, we could additionally use spatial information of the re-entrant wave front to (e.g. rotor tip tracking, local electrogram) to inform our analysis.

## 5. Supporting information

Please visit Github project **/**dblueeye/atrial-fibrillation-cellular-automata for a working implementation and for movie URLs on YouTube.
